# Antimicrobial Resistance among Neonates with Bacterial Sepsis and Their Clinical Outcomes in a Tertiary Hospital in Kathmandu Valley, Nepal

**DOI:** 10.3390/tropicalmed6020056

**Published:** 2021-04-20

**Authors:** Bijendra Raj Raghubanshi, Karuna D. Sagili, Wai Wai Han, Henish Shakya, Priyanka Shrestha, Srinath Satyanarayana, Bal Man Singh Karki

**Affiliations:** 1 KIST Medical College and Teaching Hospital, Lalitpur 44700, Nepal; henishshakya@gmail.com (H.S.); drbalmansingh@gmail.com (B.M.S.K.); 2 International Union Against Tuberculosis and Lung Disease, South East Asia Office, New Delhi 110016, India; ksagili@theunion.org (K.D.S.); ssrinath@theunion.org (S.S.); 3 Medical Statistics Division, Department of Medical Research, Ministry of Health and Sports, Yangon 15011, Myanmar; waiwai.han84@gmail.com; 4 WHO Health Emergencies Programme, Kathmandu 108, Nepal; priyankasth23@gmail.com

**Keywords:** antimicrobials, culture and drug sensitivity test, culture-confirmed sepsis, neonatal sepsis, antimicrobial resistance, LAMA

## Abstract

Globally, antibiotic resistance in bacteria isolated from neonatal sepsis is increasing. In this cross-sectional study conducted at a medical college teaching hospital in Nepal, we assessed the antibiotic resistance levels in bacteria cultured from neonates with sepsis and their in-hospital treatment outcomes. We extracted data of neonates with sepsis admitted for in-patient care from June 2018 to December 2019 by reviewing hospital records of the neonatal intensive care unit and microbiology department. A total of 308 neonates with sepsis were admitted of which, blood bacterial culture antibiotic sensitivity reports were available for 298 neonates. Twenty neonates (7%) had bacteriologic culture-confirmed neonatal sepsis. The most common bacterial species isolated were *Staphylococcus aureus* (8), followed by coagulase-negative *Staphylococcus* (5). Most of these bacteria were resistant to at least one first-line antibiotic used to manage neonatal sepsis. Overall, there were 7 (2%) deaths among the 308 neonates (none of them from the bacterial culture-positive group), and 53 (17%) neonates had left the hospital against medical advice (LAMA). Improving hospital procedures to isolate bacteria in neonates with sepsis, undertaking measures to prevent the spread of antibiotic-resistant bacteria, and addressing LAMA’s reasons are urgently needed.

## 1. Introduction

Neonatal sepsis, a bloodstream infection of newborns is a major public health problem worldwide, but it is more prevalent in low- and middle-income countries. This generally occurs in the first four weeks of life and can be either early-onset (within 1st week) or late-onset (week 1–4). Globally, about 400,000 neonates die due to sepsis [1], which is about 26% of all neonatal deaths. About 99% of these neonatal deaths take place in developing nations [2]. South Asia and Sub-Saharan Africa have the highest burden of neonatal sepsis in the world. Of the total sepsis-related neonatal deaths in 2013, 38.9% occurred in South Asia [3,4]. In Nepal, the neonatal mortality rate (NMR) is 21/1000 live births, and neonatal sepsis contributed to 16% [5]. By 2035, WHO targets reducing the NMR to 10 or fewer deaths for every 1000 live births [6]. An economic analysis of neonatal sepsis in Sub-Saharan Africa predicted a financial burden of about $9.3–469 billion annually due to neonatal sepsis and reported 5.29–8.73 million disability-adjusted life years (DALYs) could be averted by successful treatment [7]. One study done in Nepal showed that the median total cost of care of neonates admitted to Neonatal intensive care unit (NICU) was USD 222.66. The cost for sepsis was USD 226.30, and in non-sepsis, 174.02 [8].

A review of community-based studies on neonatal sepsis in low- and middle-income countries during the Millennium Development Goals (MDG) era reported that about 170 neonates per 1000 live births are clinically diagnosed for neonatal sepsis in which five neonates per 1000 live births were culture-confirmed [9]. Several non-infectious conditions resemble neonatal sepsis, especially in preterm infants, and hence clinical diagnosis is difficult. Despite this, most neonatal sepsis cases are diagnosed clinically and first-line treatment initiated. Blood culture is the standard test to confirm neonatal sepsis; it also provides an antimicrobial resistance pattern. A culture confirmation may come within 24–48 h, and the antimicrobial resistance pattern report takes 72 h. In many samples, bacterial growth is absent despite clinical symptoms [6,7]. Several hospital-based studies in Nepal reported culture-positive neonatal sepsis in the range of 2.3% to 4.2% [10,11,12]; however, some reported a much higher rate ranging between 14% to 22.4% [13]. Low birth weight and preterm babies were significantly associated with neonatal sepsis [14].

Various microorganisms are associated with early-onset sepsis (EOS) and late-onset sepsis (LOS). Group B *Streptococcus* [GBS], *Escherichia coli* [*E. coli*], *Staphylococcus aureus* [*S. aureus*], coagulase-negative *Staphylococci* [CoNS], *Listeria monocytogenes*, and other Gram-negative bacteria are generally associated with EOS. In LOS, *Klebsiella* spp. and CoNS are the primary pathogens responsible for half of the episodes. Others are *E. coli*, *Acinetobacter* spp., *Enterobacter* spp., *Candida* spp., *S. aureus*, *Enterococcus* spp., and *Pseudomonas aeruginosa* [15].

Emerging drug resistance against commonly used antimicrobials is a potential future pandemic. Like other infectious diseases, it is also posing a significant challenge in the management of neonatal sepsis. WHO estimates more than 200,000 neonatal sepsis deaths worldwide each year due to drug-resistant pathogens [1]. Studies from India and Egypt also revealed that 70% to 80% of deaths due to neonatal sepsis were due to multidrug-resistant organisms [16,17]. In recent days, microorganisms seems to be resistant against commonly used antibiotics. *S. aureus* was reported to be highly resistant against ampicillin. Gram-negative bacteria seem to be resistant against gentamicin etc.

Nepal’s demographic and health survey showed that 85% of total neonatal deaths were due to neonatal sepsis in 2011, which was higher than previous surveys, 70% in 2006 and 69% in 2001 [18]. The neonatal mortality rate (NMR) is higher in rural areas (34 deaths per 1000 live births) than in urban areas (23 deaths per 1000 live births). Currently, the emergence of multidrug-resistant bacteria poses challenges in the treatment of neonatal sepsis [19]. Neonatal infections with extended-spectrum β-lactamase [ESBL]-producing *Enterobacteriaceae* and carbapenem-resistant *Enterobacteriaceae* [CRE] are being increasingly reported. The emergence and spread of ESBL and CRE have inevitably led to the reintroduction of old antibiotics, such as colistin and polymyxin. Appropriate choice of antimicrobial agent is essential in preventing multidrug resistance and reducing morbidity and mortality [20].

This study aimed to determine the prevalence of neonatal bacterial sepsis in the KIST Medical College, the turnaround time for bacterial blood culture reports, antibiotic resistance pattern, and the clinical outcomes (hospital exit outcomes) of the neonates with suspected bacterial sepsis.

## 2. Materials and Methods

### 2.1. Study Design

This is a hospital-based cross-sectional observational study using routine data from the clinical records and laboratory registers of the Neonatal Intensive Care Unit and Clinical and Laboratory Services department of KIST Medical College, Kathmandu, Nepal.

### 2.2. Setting

#### 2.2.1. General Setting

Nepal is a landlocked country in South Asia and located mainly in the Himalayas, with an estimated population of 26.4 million. It is the 48th largest country by population and 93rd largest country by area. Kathmandu Valley is the most populated and developed place in Nepal. Lalitpur is one of the districts in Kathmandu Valley [21].

#### 2.2.2. Specific Setting

KIST Medical College is located in Mahalaxmi municipality, in the northeastern part of the Lalitpur district of Kathmandu Valley, serving 62,172 people [22]. It is a private institution with 500 beds and equipped with state-of-the-art infrastructure. Approximately 1500 deliveries are conducted at the hospital, and about 300 neonates are admitted to the neonatal ward annually.

#### 2.2.3. Laboratory Testing of Microorganisms

Blood cultures for organism’s identification and antimicrobial susceptibility tests are done by following standard Clinical and Laboratory Standard Institute (CLSI) guidelines [23]. One milliliter of blood was collected in 9 mL of brain heart infusion (BHI) broth and cultured for 24 h at 37 °C. Blood agar and MacConkey agar were incubated aerobically, whereas chocolate agar was incubated in a candle jar. If bacterial growth was observed, they were reported at 48 h and processed to identify bacterial isolates and for antimicrobial susceptibility test. In the case of no bacterial growth, they were re-incubated, and the reporting done after 72 h. Any growth of organisms was identified by standard biochemical tests followed by the Kirby Bauer disc diffusion method antimicrobial susceptibility testing. If there was no growth, a preliminary report was dispatched after 72 h and the final report after 7 days [23]. If no growth, then discarded. Culture media and antibiotic discs were obtained from HiMedia Laboratories Pvt. Ltd. (Mumbai, India).

### 2.3. Study Population and Duration

All the neonates delivered at KIST Medical College and Teaching Hospital (KIST MCTH) admitted to the Neonatal intensive care unit with clinical suspicion of sepsis from June 2018 to December 2019 were included.

### 2.4. Management of Neonatal Sepsis in KIST MCTH

At KIST MCTH, most of the neonates suspected of sepsis were managed with intravenous ampicillin and gentamicin. However, amikacin and cefotaxime were also used as first-line drugs in some cases due to increasing bacterial resistance towards ampicillin and gentamicin. Cefotaxime is also used as a first-line drug in suspected meningitis. Blood samples were sent for laboratory investigations like complete blood count, C-reactive protein (CRP), quantitative CRP, peripheral blood smear (PBS), micro erythrocyte sedimentation rate (ESR), and blood culture. Antibiotics were subjected to change as per the culture and drug sensitivity test (CDST) result. Neonates were discharged after recovery. Other expected outcomes are discharged on request, left against medical advice (LAMA) or death due to complications.

### 2.5. Definitions

#### 2.5.1. Suspected Sepsis

Irrespective of clinical symptoms, neonates with the presence of fetal or maternal risk factors were suspected for sepsis [24].

Fetal risk factors for sepsis: premature birth, low birth weight, low level of maternal IgG in preterm babies, fetal distress, low Appearance, Pulse, Grimace, Activity and Respiration (APGAR) score, resuscitation of the baby, and multiple pregnancies increase the risk of early-onset sepsis, whereas invasive procedures, such as frequent blood sampling, intubation, mechanical ventilation, catheter/probe insertion, insufficient breastfeeding, long-term parenteral nutrition, low stomach acid, and surgical interventions especially increase the risk of late-onset sepsis in the baby and maternal risk factors like chorioamnionitis, premature rupture of membranes (>18 h), intrapartum maternal fever (>38 °C).

Maternal risk factors for sepsis: delivery earlier than 37 weeks of gestation, maternal group B streptococcal (GBS) colonization, and other conditions that increase the risk of GBS infection etc., or findings suggesting sepsis in follow-up.

#### 2.5.2. Clinical Sepsis

Clinical and laboratory findings are present, but the failure to show the causative microorganism.

#### 2.5.3. Proven Sepsis

Clinical and laboratory findings are present, and demonstration of the pathogenic microorganism in cultures taken from the sterile site.

### 2.6. Data Collection, Analysis, and Statistics

We extracted the neonatal hospital records’ data from the NICU register and lab register to a paper-based proforma that included all the needed variables (Annexure 1). Data were then double-entered on a structured proforma using EpiData entry software version 3.1 (EpiData Association, Odense, Denmark). The entered data were validated and cleaned before using for analyzed. Data analysis was carried out using EpiData analysis version V2.2.2.183 (EpiData Association, Odense, Denmark).

We conducted a descriptive data analysis and summarized the neonates’ baseline demographic, perinatal, and clinical characteristics with clinically suspected sepsis using frequency and percentages. We also summarized the number and proportion with CDST results, bacteriologically confirmed sepsis, and resistance to at least one first-line antibiotic. Besides, we also summarized the bacteriological profile and antimicrobial resistance pattern disaggregated by bacterial subtype. Due to the low numbers of culture-confirmed cases, we did not attempt to study its association with neonatal parameters.

### 2.7. Ethics Considerations

The KIST Medical College administration permitted using the hospital data, and ethics approval was obtained from the Institution Ethical Board (Approval no: IRC NO:2076/77/13). Ethics approval was also sought from the Ethics Advisory Group, International Union against Tuberculosis and Lung Disease, Paris, France (Approval no: EAG 62/19).

#### Data Confidentiality

The database did not include any personal identifiers, and a unique identifier was assigned for each record. The hard copies of the data proforma were kept in locked cabinets after data entry. The electronic databases were stored in a password protected computer of the principal investigator.

## 3. Results

### 3.1. Characteristics of Neonates Admitted with Suspected Sepsis at KIST MCTH

**Figure 1:**
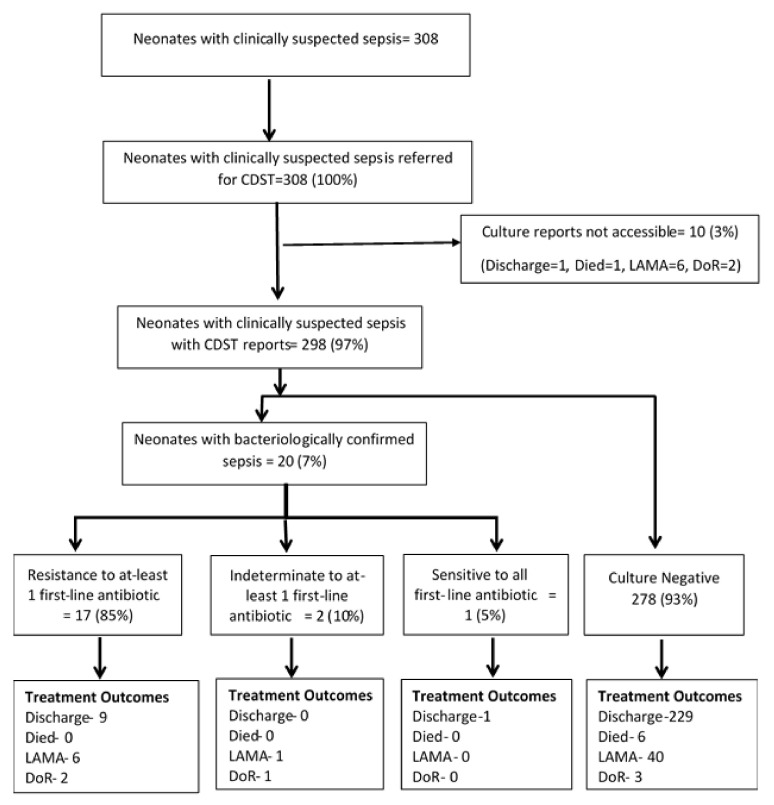
Flow-chart depicting uptake of CDST, bacteriological confirmation, resistance to first-line drugs, and treatment outcomes among neonates with clinically suspected sepsis admitted to NICU at KIST MCTH, Kathmandu Valley, Nepal during June 2018–December 2019. CDST—Culture and drug susceptibility test; NICU—neonatal intensive care unit; LAMA—left against medical advice; DoR—discharge on request; First-line antibiotics—ampicillin, amikacin, gentamicin, and cefotaxime.

During the study period (i.e., June 2018 to December 2019), a total of 1102 neonates were admitted to the NICU of KIST MCTH, of which 308 (28%) were suspected of neonatal sepsis. Of the suspected cases, 298 (93%) had bacterial CDST reported available. The majority of the suspected neonates were bacterial culture-negative for sepsis, with only 7% having culture-confirmed sepsis. Out of these, only one neonate had bacteria sensitive to all antibiotics tested, whereas the remaining 19 neonates were associated with resistance to at least one first-line antibiotic. Figure 1 presents the neonates admitted to NICU with clinically suspected sepsis and culture-confirmed cases and their clinical outcomes.

The demographic, perinatal, and clinical characteristics of the neonates are shown in Table 1. The majority of clinically suspected neonatal sepsis cases (i.e., 91%) were aged <3 days. Similarly, the majority of the culture-confirmed cases were also from the same age group. There were slightly more male neonates than female neonates (56% vs. 46%). Most of the neonates were delivered vaginally; however, less than half were full term. Nearly 40% had clinical jaundice, and a majority (87%) had low c-reactive protein levels.

### 3.2. Turnaround Time of Bacterial CDST and Change in Antibiotic after CDST Results

Table 2 shows the turnaround time of bacterial CDST and change in antibiotic after CDST results. Blood samples for bacterial cultures were requested for all the neonates at the time of admission, and the results were available for 97%. A majority (87%) of the culture and sensitivity reports were received within three days. In 32 neonates, antibiotics were changed during treatment; however, this was irrespective of blood culture reports.

### 3.3. Antibiotic Resistance Pattern of Isolates

Table 3 shows the antibiotic resistance pattern of the isolates. A total of 20 neonates had bacteriologically confirmed sepsis. Of which, 14 isolates were Gram-positive bacteria, and six were Gram-negative. *Staphylococcus aureus* was the most common isolate, followed by coagulase-negative *Staphylococcus*. Isolated bacteria were mostly resistant to first-line antibiotics like ampicillin, cefotaxime, moderately resistant to ciprofloxacin, cotrimoxazole, and chloramphenicol and highly sensitive to imipenem, meropenem, and vancomycin. None of the tested isolates of Gram-positive bacteria showed resistance to vancomycin.

It is noted that isolates were tested for second-line drugs only if the treating physicians request or resistance to 3 or more than three 1st line antibiotics. Hence it is not possible to determine the complete resistance pattern for the second-line drugs.

### 3.4. Treatment Outcome of Clinically Suspected Sepsis Neonates

**Figure 2:**
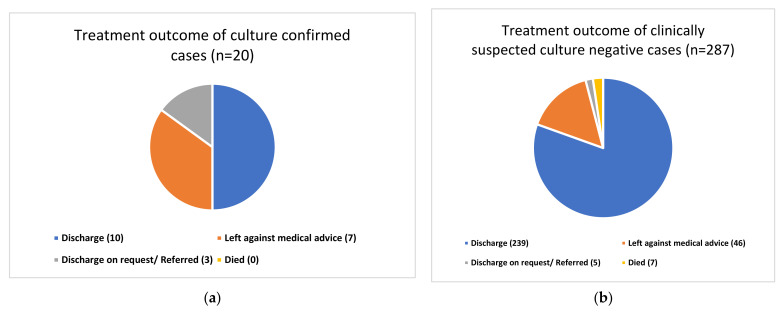
(**a**) Treatment outcome of culture-confirmed cases; (**b**) Treatment outcome of clinically suspected culture-negative cases.

The treatment outcome of all neonates suspected of sepsis disaggregated by bacterial growth status is shown in Figure 2a,b. Only seven hospital deaths were observed in the study, all of them in the culture-negative group. A majority (n = 240) of the remaining were discharged from the hospital, with quite a few of them leaving the hospital against medical advice.

## 4. Discussion

The present study documents 28% clinically suspected neonatal sepsis in the Lalitpur district of Kathmandu Valley, Nepal. Culture positivity among this neonatal population was observed to be low (7%). Previous studies from Nepal have reported in the range as low as 2.3% to as high as 37% [11,13,25]. The difference in the culture positivity could be due to differences in culture methods used, the amount of blood volume collected, the timing of blood culture, culture technique, and others. A study from a different region (Iran) had also observed a similar culture positivity rate (6.6%) [17]. Nonetheless, a high proportion of culture-negative sepsis was treated with broad-spectrum antibiotics, which is of great concern for antibiotic resistance development. An internal evaluation of the care cascade for suspected neonatal sepsis at the study site would help identify any improvement areas.

Most clinically suspected neonatal cases were early-onset cases (91%), similar to other studies in the region [11]. However, some studies have reported a higher proportion of late-onset sepsis [25]. In our study, most of the culture-confirmed cases were early-onset cases (75%). Though there were a high proportion of male children admitted, it was not statistically significant. Early-onset sepsis is associated with high mortality among neonates worldwide, and it is crucial to strengthen the hospital’s diagnosis and care for early-onset sepsis [26].

In our study setting, culture reports’ turnaround time was mostly three days (87%). However, a few reports were not available or missing, which should be of concern. Additionally, the hospital registration number is not documented in the laboratory database, making it difficult to trace the reports. To improve this situation, the hospital has initiated an electronic database system since late 2019, which is encouraging.

Of the 20 culture-confirmed cases, the common causative organism was Gram-positive *S. aureus* (40%), followed by CoNS (25%). In Gram-negative, 15% were *Acinetobacter* species, and 15% were *Klebsiella*. A study done by Bhattarai et al. showed [25] *S. aureus* as the most common organism (34%), followed by *Klebsiella* (32%) and CoNS (23%). This is almost similar to our study. Organisms like *Klebsiella*, *Acinetobacter* are generally associated with healthcare-associated infection, which is usually found in late-onset neonatal sepsis. A recent study by Shrestha et al. [13] also showed *Klebsiella* as the most common (48%), followed by CoNS (17%). In another study done in a tertiary care center in Kathmandu, Nepal [11], CoNS was the most commonly isolated bacteria (42.85%), followed by *S. aureus* (29.41%). In a study by Yadav et al. [27], the predominant organisms identified were *S. aureus* (38%) and *Klebsiella pneumoniae* (31%). From these studies, we can see the variation in the causative organism pattern, but *S. aureus*, CoNS, *Acinetobacter*, *Klebsiella*, and *E. coli* are some of the most common bacteria causing neonatal sepsis in Nepal.

Even though the total number of culture-confirmed cases is small (N = 20), the study also documented 90% of all culture-confirmed cases were resistant to at least one first-line antibiotics, indicating the need for continuous surveillance of antibiotic resistance. The most common drugs to which resistance was observed were ampicillin (85%) and cefotaxime (80%), followed by ciprofloxacin (40%) and cotrimoxazole (40%). RK Shrestha et al. also reported high (91%) resistance for ampicillin among *S. aureus* [21]; however, resistance to other drugs was on the lower side. They had also reported a 62% resistance to azithromycin, which was not tested in our study. However, a study from India reported 37% resistance to ampicillin [28]. Yadav et al. [27] reported 87% of Gram-negative and 80% of *S. aureus* as resistant to ampicillin. Gentamicin resistance among Gram-negative and *S. aureus* was 50% and 80%, respectively. The prevalence of ciprofloxacin resistance was 20% among *S. aureus* and 13% among Gram-negative bacilli [20].

Regarding the clinical outcomes of the neonates, most of them were discharged after recovery. No deaths were observed among the culture-confirmed cases; however, six deaths were observed in the culture-negative group, and overall, seven deaths (2%) were recorded. This is very low compared to most of the studies. Pokhrel et al. reported 16% mortality among culture-confirmed sepsis in a tertiary hospital in Nepal [29]. A study from Bhutan reported 26 deaths, of which 8 deaths were due to, which was 30.8% of all mortality and 88.9% of all culture-positive sepsis deaths [30].

About 17% (53/308) of the cases were documented to have left the hospital against medical advice (LAMA). This appeared to be very high—the reasons for the same need to be explored further. The reasons could be either provider-related or patient-related, or both. For instance, KIST MCTH is a private medical college; hence, the cost of services at NICU may be a reason. Since, the majority of people using the services at KIST are from a lower socio-economic background, this may be the most probable reason. However, KIST is a medical college and the costs are nominal compared with other private hospitals. The current study did not look at the reasons. Future studies to explore the reasons may help the hospital administration to improve their services for patient benefit.

The study’s main limitations were: (a) data for the year 2018 were available only for seven months, a reason for the low number of study participants; (b) the culture positivity observed in the study is low compared to other studies in Nepal. The reasons for this was not studied and is an area for future research; (c) the outcomes reported here are hospital exit outcomes. If there were any further complications following exit from the hospital, our study does not provide information on this aspect. Not many previous studies from Nepal reported on the hospital exit outcomes.

Despite the limitations, a few learnings from this study can improve neonatal sepsis management in the hospital. These include (a) improving the recording and reporting system in the hospital, especially between the NICU and the laboratory, to ensure that all culture samples are received, and reports dispatched on time; (b) treatment of a large number of culture-negative neonates with broad spectrum of antibiotics is an area of concern, efforts need to be made to improve culture positivity; (c) the in-hospital mortality rate was low which indicates good management of the neonatal sepsis; however a large number of cases leaving against medical advice should be investigated further to understand the reasons and measures to address them; (d) it was observed that most often the drug sensitivity tests are not conducted on all the second-line drugs, and only conducted on request from the physician or when isolates are resistant to three or more than three first-line drugs. There needs to be consistent testing for the resistance to all the drugs to enable monitoring and management of antibiotic resistance.

## 5. Conclusions

In conclusion, the growth of bacteria in blood samples of neonates treated for sepsis in KISH MCTH was low but the isolated bacteria showed high resistance to ampicillin and cefotaxime. The treatment outcomes were good with 78% getting discharged after successful resolution of neonatal sepsis. Improving hospital procedures to isolate bacteria in neonates with sepsis, undertaking measures to prevent the spread of antibiotic-resistant bacteria, and addressing reasons neonates leaving hospital against medical advice are urgently needed to improve the quality of care in this hospital.

## Figures and Tables

**Table 1:** Demographic, perinatal, and clinical characteristics of neonates with clinically suspected sepsis, culture-confirmed, and those with resistance to at least one first-line antibiotic admitted to NICU at KIST MCTH, Kathmandu Valley, Nepal during 2018–2019.

Characteristics	Clinically Suspected N = 308	Culture Confirmed N = 20	Resistant to at Least One First-Line Antibiotic N = 18
	N (%)	N (%)	N (%)
**Demographic**			
**Age in days**			
≤3	280 (91)	15 (75)	13 (72)
4–7	14 (4.5)	4 (20)	4 (22)
>8	14 (4.5)	1 (5)	1 (6)
**Gender**			
Male	172 (56)	11 (55)	9 (50)
Female	133 (43)	8 (40)	8 (44)
Missing	3 (1)	1 (5)	1 (6)
**Year of admission**			
2018 (June–December)	116 (38)	10 (50)	10 (56)
2019 (January–December)	192 (62)	10 (50)	8 (44)
**Perinatal characteristics**			
**Gestational age**			
Preterm (<37 w)	46 (15)	4 (20)	4 (22)
Early-term (37–38 w + 6 d)	68 (22)	8 (40)	10 (55)
Full-term (39−40 w + 6 d)	136 (44)	5 (25)	2 (11)
Late-term (40−41 w + 6 d)	32 (10)	0 (0)	0 (0)
Post-term (>42 w)	7 (2)	1 (5)	1 (6)
Missing	18 (6)	2 (10)	1 (6)
**Mode of delivery**			
Normal	162 (52)	10 (50)	9 (50)
Cesarean section	141 (46)	8 (40)	7 (39)
Missing	5 (2)	2 (10)	2 (11)
**Birth weight in grams**			
Very low (<1500 gm)	6 (2)	0 (0)	0 (0)
Low (1501–<2500 gm)	55 (18)	5 (25)	5 (28)
Normal (≥2500 gm)	247 (80)	15 (75)	13 (72)
**Clinical Characteristics**			
**Jaundice**			
Negative	184 (60)	12 (60)	12 (67)
Positive	119 (39)	7 (35)	5 (28)
Missing	5 (1)	1 (5)	1 (5)
**Hyperbilirubinemia (Bilirubin level) (mg/dL)**			
≥5	4 (1)	0 (0)	0 (0)
6–15	70(23)	2 (10)	2 (11)
>15	44(14)	5 (25)	3 (17)
No	190(62)	13 (65)	13 (72)
**Baseline C-reactive protein**			
High(+ve)	33 (10)	1 (5)	1 (6)
Low(−ve)	267 (87)	18 (90)	16 (88)
Missing	8 (3)	1 (5)	1 (6)

NICU = Neonatal intensive care unit. KIST MCTH = KIST Medical College and Teaching Hospital.

**Table 2:** Turn-around time of CDST and change in antibiotic after CDST results among neonates with clinically suspected sepsis admitted to NICU at KIST MCTH, Kathmandu Valley, Nepal during 2018–2019.

Variables	All Neonates n (%)	Culture Positive Neonates n (%)
**Receipt of CDST results**		
Yes	298 (97)	20
No	10 (3)	0
**Day of receipt of CDST results after admission**		
3	269 (87)	10 (50%)
4	14 (5)	7 (35%)
5	3 (1)	3 (15%)
≥5	1 (0)	-
Missing	2 (1)	-
**Antibiotic change made**		
Yes	32 (10)	3 (15%)
No	18 (6)	17 (85%)
Not applicable	258 (84)	-

CDST = culture and drug-sensitive test. NICU = neonatal intensive care unit. KIST MCTH = KIST Medical College and Teaching Hospital.

**Table 3:** Profile and antimicrobial resistance pattern of Gram-positive isolates (N = 14) and Gram-negative isolates (N = 6) among neonates with bacteriologically confirmed sepsis admitted to NICU at KIST MCTH, Kathmandu Valley, Nepal during 2018–2019 (R = resistance).

	Gram-Positive Isolates			Gram-Negative Isolates	
Drugs	*S. aureus*	CoNS	*Enterococcus* Species	*Acinetobacter* Species	*Klebsiella pneumoniae*
**Total isolates**	8	5	1	3	3
**1st line antibiotics (no. of resistance/no. of isolates tested)**					
Amikacin (6/18)	1	2	1	1	1
Ampicillin (17/18)	7	4	1	2	3
Cefotaxime (16/19)	7	3	1	2	3
Ceftriaxone (6/14)	2	1	1	1	1
Ciprofloxacin (8/18)	2	2	1	1	2
Cotrimoxazole (8/19)	3	1	1	1	2
Chloramphenicol (4/15)	1	0	0	2	1
Carbenicillin (1/1)	0	0	0	1	0
Gentamycin (1st) (6/18)	1	2	1	1	1
Piperacilin + tazobactam (1/1)	0	0	0	1	0
**2nd line antibiotics**					
Amoxicillin-clavulanic acid (5/6)	1	0	0	2	2
Ceftazidime (6/6)	1	0	1	2	2
Cefepime (3/4)	1	0	0	1	1
Imipenem (0/4)	0	0	0	0	0
Meropenem (2/10)	0	0	1	1	0
Ofloxacin (4/5)	1	0	1	1	1
Tetracycline (0/1)	0	0	0	0	0
Vancomycin (0/4)	0	0	0	0	0

## Open Access Statement and Disclaimer

In accordance with WHO’s open-access publication policy for all work funded by WHO or authored/co-authored by WHO staff members, the WHO retains the copyright of this publication through a Creative Commons Attribution IGO license (http://creativecommons.org/licenses/by/3.0/igo/legalcode, accessed on 8 April 2021) which permits unrestricted use, distribution and reproduction in any medium provided the original work is properly cited. In any use of this article, there should be no suggestion that WHO endorses any specific organization, products or services. The views expressed in this article are those of the authors and do not necessarily reflect those of their affiliated institutions. The use of the WHO logo is not permitted. This notice should be preserved along with the article’s original URL.
